# Protein S Enhances the Phagocytosis of Phosphatidylserine‐Exposing Erythrocytes: Implications in Sickle Cell Disease

**DOI:** 10.1002/ajh.70117

**Published:** 2025-10-25

**Authors:** Claire Auditeau, Aurélie Fricot, Raphaël Gauthier, Abdoulaye Sissoko, Zeynep Cacan, Céline Gounou, Laure Joseph, Sandra Manceau, Slimane Allali, Michael Dussiot, Mickael Marin, Alexis Lavergne, Mariem Khamari, Sophie Moog, Elsa Bianchini, Laetitia Claer, Sandrine Laurance, Valentine Brousse, Sébastien Eymieux, Philippe Roingeard, Pascal Amireault, Thiago Trovati Maciel, Alain Brisson, Pierre Buffet, François Saller, Delphine Borgel, Camille Roussel

**Affiliations:** ^1^ Hémostase, Inflammation, Thrombose (HITh), UMR‐S1176, INSERM Université Paris‐Saclay Le Kremlin‐Bicêtre France; ^2^ Service D'hématologie Biologique Hôpital Necker Enfants Malades, Assistance Publique‐Hôpitaux de Paris (AP‐HP) Paris France; ^3^ Université Paris Cité, INSERM, BIGR Paris France; ^4^ UMR‐CBMN, CNRS‐Université de Bordeaux‐IPB Pessac France; ^5^ Department of Biotherapy, French National Sickle Cell Disease Referral Center Hôpital Necker, Assistance‐Publique Hôpitaux de Paris, Gr Ex Paris France; ^6^ Reference Centre for Sickle Cell Disease Necker‐Enfants Malades University Hospital, AP‐HP Centre Paris France; ^7^ Laboratory of Cellular and Molecular Mechanisms of Hematological Disorders and Therapeutic Implications, INSERM, Institut Imagine Université Paris Cité Paris France; ^8^ Inovarion 251 Rue Saint Jacques Paris France; ^9^ Centre de Référence MCGRE, Service D'hémato‐Immunologie Hôpital Universitaire Robert Debré, APHP Paris France; ^10^ IBiSA Electron Microscopy Facility University Hospital Center of Tours Tours France; ^11^ Inserm U1259 MAVIVHe Université de Tours and CHRU de Tours Tours France; ^12^ Service de Maladies Infectieuses et Tropicales Hôpital Universitaire Necker Enfants Malades Université Paris Cité Paris France

**Keywords:** erythrocyte ghosts, phagocytosis, PROS1, protein S, sickle cell disease

## Abstract

The major anticoagulant Protein S (PROS1) also contributes to the phagocytosis of apoptotic cells by bridging exposed phosphatidylserine (PtdSer) to the MerTK receptor on macrophages (efferocytosis). Whether PROS1 is involved in the splenic clearance of PtdSer‐positive senescent and altered erythrocytes such as erythrocyte ghosts (eryghosts) is unknown. Here, we investigate the contribution of PROS1 and MerTK to the phagocytosis of intact RBC and eryghosts in healthy subjects and patients with sickle cell disease (SCD). We show that PROS1 enhances the phagocytosis of ionomycin‐treated PtdSer‐positive erythrocytes and of eryghosts generated in vitro. We confirm that eryghosts circulate in patients with SCD at higher levels than in healthy subjects and observe increased hemolysis and decreased levels of plasmatic PROS1 in patients with the highest concentration of eryghosts. The proportion of circulating eryghosts is correlated with the intensity of hyposplenism, and eryghosts are less frequently observed in sections of SCD compared to control spleens. We demonstrate that circulating eryghosts are procoagulant and adhere to endothelial cells. In SCD, PROS1 enhances their phagocytosis in a MerTK‐dependent manner but has no such effect on intact erythrocytes. PROS1 is therefore involved in erythrophagocytosis, a physiological process insufficient in patients with SCD due to intense intravascular hemolysis and hyposplenism, leading to PROS1 consumption and the abnormal persistence of eryghosts in circulation. PROS1 deficiency may in turn initiate a pathogenic loop, enhancing unregulated activation of coagulation and defective clearance of procoagulant and adherent eryghosts. This deeper understanding of physiological and pathological erythrophagocytosis opens new therapeutic approaches targeting PROS1 in SCD.

## 1 Introduction

Protein S (PROS1) is a vitamin K–dependent protein that enhances two major anticoagulant pathways by acting as a nonenzymatic cofactor for activated protein C (APC) [1] and tissue factor pathway inhibitor α (TFPIα) [2, 3]. Besides anticoagulation, PROS1 is implicated in various cellular processes through its binding to tyrosine kinase receptors MerTK and Tyro3, which are members of the TAM receptor family [4]. Notably, efferocytosis [5], e.g., the phagocytosis of apoptotic cells by macrophages, is mainly mediated by MerTK, a receptor expressed primarily on macrophages [6]. PROS1 serves as a bridging molecule between MerTK‐expressing macrophages and phosphatidylserine (PtdSer)‐exposing apoptotic cells [5]. PtdSer is usually found on the inner leaflet of the cell membrane and its exposure on the apoptotic cell surface is an “eat me” signal for phagocytes [7, 8]. The amino‐terminal γ carboxyglutamic acid (GLA) domain of PROS1 binds with high affinity to PtdSer exposed by apoptotic cells, while its carboxy‐terminal region comprising the sex hormone–binding globulin‐like (SHBG‐like) domain enables MerTK binding and activation [5]. Although PtdSer exposure is a recognized signal for clearance of senescent and pathogenic red blood cells (RBC) [9] by the spleen, the putative role of PROS1 and MerTK in this mechanism has never been studied. In 2020, Klei et al. showed that intrasplenic hemolysis contributes to RBC clearance. They observed that retention of senescent RBC in the spleen results in their hemolysis and the formation of RBC ghost shells [10]. During this hemolytic process, these RBC become permeable to hemoglobin, resulting in a cell‐like structure devoid of hemoglobin, called erythrocyte ghosts or eryghosts [11]. These eryghosts were prone to recognition and clearance by red pulp macrophages (RPM) in the spleen [10], yet the mechanism leading to this recognition remains elusive. Although infrequent, circulating eryghosts were isolated from the plasma of healthy individuals [12] and were found to massively expose PtdSer. In addition, PtdSer‐exposing eryghosts were observed in a higher proportion in the peripheral blood of patients with sickle cell disease (SCD) [13, 14], in which PROS1 levels are frequently decreased [15, 16]. The intense PtdSer exposure of eryghosts led us to hypothesize that PROS1 is implicated in the clearance of RBC in physiology and disease by acting as a bridging molecule that leads to eryghost recognition and clearance by RPM in the spleen. We also hypothesized that this pathway is insufficient in SCD due to intense hemolysis and hyposplenism and leads to circulating eryghosts, participating in the pathophysiology of the disease.

## 2 Materials and Methods

Materials (reagents, antibodies, references, concentrations) are detailed in Supporting Information: Supplemental Methods.

### 2.1 Human Subjects

Human subjects without underlying RBC‐related disease and with SCD (S/S genotypes) were included at two hospitals of the Assistance Publique‐Hôpitaux de Paris (AP‐HP) network in a study entitled “Pathophysiological Explorations of Red Blood Cells” (ClinicalTrials.gov reference NCT03541525). Human spleens were retrieved in the context of the Spleenvivo project approved by the “Ile‐de‐France II” Institutional Review Board on September 4, 2017 (#CPP 2015‐02‐05 SM2 DC).

All patients with SCD were in a non‐crisis “steady state,” none had received blood products within 1 month.

#### 2.1.1 Blood Collection

Blood was drawn into lithium heparin collection tubes (for whole blood analysis) or citrated tubes with 3.2% sodium citrate (1:9) (for plasma analysis). Platelet‐free plasma (PFP) was prepared by two successive centrifugation steps at 2500 g for 15 min each and frozen at −80°C until use [17].

#### 2.1.2 Spleen Collection

Spleens were retrieved and processed as previously described [18]. Spleens were collected within 2 h of surgery on the day of splenectomy. Normal spleens were from patients undergoing distal splenopancreatectomy for pancreatic disease. In all cases, these normal spleens had no macroscopic or microscopic abnormalities. Patients with SCD had partial or total splenectomy for acute spleen sequestration crisis/hypersplenism. Of note, these patients received a transfusion prior to the surgery.

### 2.2 Generation, Isolation and Identification of Eryghosts

#### 2.2.1 Generation of Eryghosts

Normal RBC were incubated with 5P8 (5 mM NaH_2_PO_4_/Na_2_HPO_4_, 0.05 mM EDTA, pH 8) at a 2.5% hematocrit to induce an osmotic shock resulting in hemolysis. Then, centrifugation steps (15,000 g for 15 min) were performed until a white pellet was formed. Eryghosts were resuspended in DPBS (Dulbecco's Phosphate Buffer Saline) and characterized using imaging flow cytometry.

#### 2.2.2 Isolation and Quantification of Eryghosts From the Platelet‐Free Plasma of Patients With SCD

Although eryghosts are similar in size to RBC and therefore larger than platelets, they were shown to be present in PFP [12]. This observation is likely due to their minimal cytoplasmic content, which makes them lighter and less prone to sedimentation. In contrast, platelets and RBC retain a dense cytoplasm, promoting sedimentation. Flow cytometry data (not shown) indicate that eryghosts exhibit lower light‐scatter properties than platelets and RBC, consistent with their reduced internal complexity. Frozen PFP was thawed for 5 min at 37°C and gently mixed. The quantification of eryghosts in PFP was performed by flow cytometry [19]. Circulating eryghosts were isolated by centrifuging the PFP of patients with SCD or healthy volunteers at 3000 g for 15 min.

#### 2.2.3 Identification of Eryghosts Using Imaging Flow Cytometry

Imaging flow cytometry was used to identify eryghosts in various samples (in vitro eryghosts suspension, whole blood, PFP). Eryghosts were identified as cells with a ghost aspect in brightfield due to the lack of hemoglobin content (60× magnification) and a positive anti‐glycophorin A (GPA) staining. Imaging flow cytometry was performed using ImageStream X Mark II, Amnis Flow Cytometry (part of Cytek Biosciences, Seattle, WA, United States). Images were processed with computer software IDEAS [version 6.3] (Amnis). Single‐stain controls were run for the different fluorochromes used, and spectral compensation was performed.

### 2.3 Protein S Detection at the Surface of Circulating Eryghosts

For the detection of PROS1 on circulating eryghosts, whole blood was diluted at 1% hematocrit in HBSS (Hanks Balanced Saline Solution) containing 5 mM CaCl_2_ and 1% albumin. An anti‐protein S antibody was incubated with diluted whole blood for 1 h at RT and detected by an Alexa Fluor 568‐conjugated goat anti‐Rat IgG. The same experiment was performed after a 1‐h incubation at 37°C with 100 nM purified plasma‐derived human PROS1.

### 2.4 Quantification of RBC and Eryghosts Exposing Phosphatidylserine

For the detection of PtdSer exposure, cells were diluted 1:1000 in DPBS before being incubated with FITC‐labeled bovine lactadherin for 15 min at room temperature (RT). Flow cytometry was performed using FACscan analysis on FACSCanto II (BD Biosciences).

### 2.5 Phagocytosis Assay

Target cells including ionomycin‐treated RBC (iono‐RBC, prepared as described [20]), intact RBC, and eryghosts were fluorescently labeled with either 5(6)‐carboxyfluorescein diacetate N‐succinimidyl ester (CFSE) or anti‐GPA antibody and suspended in 0.5 mL of culture medium (RPMI, GlutaMAX, 25 mM Hepes, 10% fetal bovine serum, 1% penicillin–streptomycin). RBC and eryghosts were either incubated for 5 min at 2.5% hematocrit with CFSE or for 30 min at 0.5% hematocrit with the anti‐GPA antibody and then washed two times with DPBS.

#### 2.5.1 THP1‐Derived Macrophages

THP‐1 monocytes were differentiated into THP‐1‐derived macrophages using phorbol 12‐myristate 13‐acetate (PMA, 20 ng/mL) added 48 h before the experiment [21]. Then, target cells (iono‐RBC, intact RBC, eryghosts) that were fluorescently labeled were incubated with the plated, adherent human macrophages for 1 or 4 h at 37°C. Macrophages were washed 5 times with DPBS to remove non‐engulfed target cells. Macrophages were detached from plates with 0.05% trypsin–EDTA for 20 min at 37°C. Ice‐cold culture medium containing 2 mM EDTA was added to stop trypsin action. Then, macrophages were scraped from the culture plate and transferred into ice‐cold DPBS‐1% albumin. This assay was performed in the presence or absence of purified plasma‐derived human PROS1 (50 or 100 nM) and UNC2025 (MerTK receptor inhibitor, 5 μM, added 30 min before the addition of target cells).

#### 2.5.2 Human Monocyte‐Derived Macrophages (hMDMs)

Monocytes were isolated from the whole blood of patients with SCD (*n* = 6) and healthy control subjects (*n* = 4). Whole blood was first subjected to Ficoll‐Paque density gradient centrifugation to separate peripheral blood mononuclear cells (PBMCs [22]) from other blood components. Monocytes were enriched from PBMCs by magnetic‐activated cell sorting using CD14 microbeads. Monocytes were differentiated into hMDMs as described by Gerlach et al. [23]. Briefly, monocytes were cultured for 6–7 days in culture medium (RPMI, GlutaMAX, 25 mM Hepes, 10% fetal bovine serum, 1% penicillin–streptomycin) supplemented with M‐CSF (50 ng/mL). Then, eryghosts fluorescently labeled by the anti‐GPA antibody were incubated with the plated, adherent hMDMs for 1 h at 37°C. Macrophages were washed five times with DPBS to remove non‐engulfed target cells, and detached from plates with accutase (undiluted) for 10 min at 37°C. Ice‐cold culture medium containing 2 mM EDTA was added to stop accutase action. Then, macrophages were scraped from the culture plate and transferred into ice‐cold DPBS‐1% albumin. This assay was performed in the presence or absence of purified plasma‐derived human PROS1 (100 nM).

In all phagocytosis assays, cytochalasin D was used as a negative control in this assay as it inhibits actin polymerization, which is mandatory for the phagocytosis process. Fluorescence intensity was quantified by flow cytometry to determine the percentages of fluorescent macrophages that had phagocytosed fluorescent target cells.

### 2.6 Pocked RBC Counts to Quantify Hyposplenism

The quantification of pocked RBC as a biomarker of impaired splenic RBC filtration was performed as previously described by Holroyde et al. [24]. Briefly, whole blood was fixed in 0.1% DPBS‐buffered glutaraldehyde. The proportion of pocked RBC was determined from at least 300 RBC using a Leica microscope (DM1000 LED model) with differential interference contrast (DIC) based on the principle of Normarski optics.

### 2.7 Transmission Electron Microscopy

#### 2.7.1 Images of Spleen Sections

The splenic biopsies were aseptically processed for histological and transmission electron microscopy (TEM) studies as previously described [18, 25] and detailed in Supporting Information: Supplemental Methods. Macrophages were identified as previously described [26].

#### 2.7.2 Images of Eryghosts Prepared In Vitro

Eryghosts were prepared in vitro as described above and were isolated using magnetic particles conjugated with Annexin V. Eryghosts were concentrated by magnetophoresis and processed for ultramicrotomy according to standard procedures, detailed in Supporting Information: Supplemental Methods. TEM observations were performed with a FEI CM120 electron microscope (FEI, USA) operated at 120 kV. Images were recorded with a USC1000 slow scan CCD camera (Gatan, USA).

### 2.8 Procoagulant Activity of Circulating Eryghosts From Patients With SCD

Normal human plasma (Cryopep) was spiked with either Hepes/BSA buffer (20 mM Hepes, 6% BSA, pH 7.4) or increasing concentrations of isolated eryghosts diluted in Hepes/BSA buffer (0.5–4.10^6^/mL). Coagulation was initiated by tissue factor. Thrombin generation and fibrin formation were measured as described [27, 28] and detailed in Supporting Information: Supplemental Methods.

### 2.9 In Vitro Dynamic Adhesion Assay of RBC and Eryghosts to Endothelial Monolayers Stimulated With TNF‐α

Circulating RBC and eryghosts from three patients with SCD were used to perform adhesion experiments on TNFα‐activated human microvascular endothelial cell line 1 (HMEC‐1), as previously described [29] and detailed in Supporting Information: Supplemental Methods. Briefly, mixtures containing RBC and eryghosts were stained with CFSE and were perfused for 10 min. After a sedimentation step, washes were performed at 0.2 dyn and 5 min at 1 dyn with HBSS‐5 mM CaCl_2_‐1% albumin to remove non‐adherent RBC and eryghosts. Images were analyzed to count adherent RBC and eryghosts. All CFSE+ cells were counted to determine the total number of adherent cells. Intact RBC were visible on the brightfield images, but eryghosts were not. Therefore, the number of adherent eryghosts was calculated as follows:

$$\begin{array}{c} \text{Eryghosts}=\text{total CFSE}+\text{cells}\\ \;–\text{intact}\;\mathrm{RBC}\;\text{counted}\;\mathrm{on}\;\text{brightfield images}. \end{array}$$

The number of adherent intact RBC and eryghosts per area of biochip was normalized on the initial proportion of each population added with the following equation:

$$\begin{array}{c} \text{Normalized}\;\mathrm{RBC}\;\text{or eryghosts}\;\mathrm{per}\;\text{area of biochip}\\ =\left(\text{number of adherent}\;\mathrm{RBC}\;\text{or eryghosts}\times 100\right)/\\ \text{initial proportion added}. \end{array}$$

$$\begin{array}{c} \text{Normalized ratio}\;\left(\text{eryghosts}/\text{intact}\;\mathrm{RBC}\right)\\ =\left(\text{adherent eryghosts}/\text{adherent}\;\mathrm{RBC}\right)/\\ \left(\text{initial proportion eryghosts}/\text{initial proportion of}\;\mathrm{RBC}\right). \end{array}$$

### 2.10 Statistics

One‐way ANOVA was used as a statistical test of variance with Tukey or Dunnett post‐test for multiple comparisons. Otherwise, the 2‐tailed paired Student's *t*‐test was used. Spearman test was used for correlations. A *p* value < 0.05 was considered statistically significant. Statistical analyses were performed using Prism software (GraphPad version 10.0.0, Boston, Massachusetts USA).

Data are expressed as mean ± standard deviation (SD).

## 3 Results

### 3.1 Protein S Stimulates Phagocytosis of Phosphatidylserine‐Exposing Erythrocytes in a MerTK‐ Dependent Manner

**FIGURE 1:**
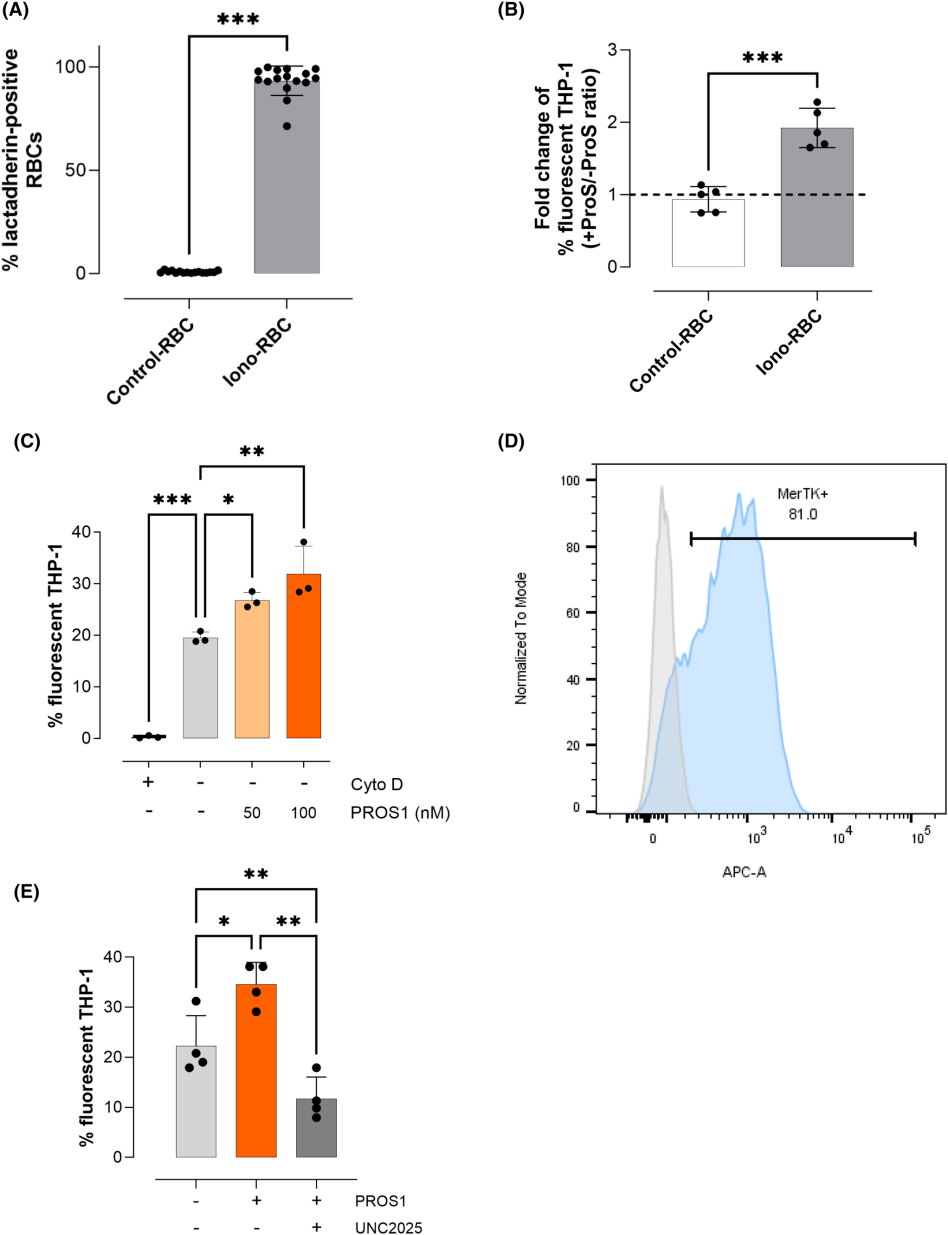
Protein S enhances the phagocytosis of ionomycin‐treated RBC in a MerTK‐dependent manner. (A) Proportion of normal (Control‐RBC, white) and ionomycin‐treated RBC (Iono‐RBC, gray) exposing PtdSer, quantified by lactadherin‐FITC staining. Results are expressed as mean ± SD of 16 independent experiments. Student t‐test is used, ***p < 0.001. (B) Effect of PROS1 on the phagocytosis of normal (Control‐RBC, white) and ionomycin‐treated RBC (Iono‐RBC, gray). The ratio between the percentage of fluorescent THP‐1 macrophages with and without supplementation with 100 nM of PROS1 after a 4‐h incubation is plotted. The black dotted line represents the absence of prophagocytic effect of PROS1 (ratio = 1). Results are expressed as mean ± SD of 5 independent experiments. Student t‐test is used, ***p < 0.001. (C) Phagocytosis of iono‐RBC by THP‐1‐derived macrophages as expressed by the percentage of fluorescent‐THP1 in the presence of cytochalasin D (negative control, black), or culture medium alone (gray), or increasing concentrations of PROS1 (50 nM light orange, 100 nM orange), results are expressed as mean ± SD of 3 independent experiments. One‐way ANOVA with Dunnett's multiple comparison tests (comparison to culture medium alone) are used. (D) MerTK expression on THP‐1 derived macrophages detected by flow cytometry. THP‐1 macrophages are incubated with (blue) and without (gray) an APC‐conjugated anti‐MerTK antibody. A representative result from 3 independent experiments is shown. (E) Phagocytosis of iono‐RBC erythrocytes by THP‐1‐derived macrophages in the absence (light gray) or presence of PROS1 (100 nM, orange), or PROS1 + UNC2025 (PROS1 100 nM, UNC2025 5 μM, dark gray), results are expressed as mean ± SD of 3 independent experiments. One‐way ANOVA with Tukey's multiple comparison tests are used, **p < 0.01, ***p < 0.001. [Color figure can be viewed at wileyonlinelibrary.com]

We evaluated the prophagocytic activity of PROS1 on ionomycin‐treated RBC (Iono‐RBC). Ionomycin was used to induce PtdSer translocation to the external surface of RBC. The proportion of Iono‐RBC exposing PtdSer was high (93.3% ± 7.1%, *n* = 16), whereas that of normal RBC not exposed to ionomycin (Control‐RBC) was very low (0.8% ± 0.5%, *n* = 16, Figure 1A). Iono‐ and Control‐RBC were fluorescently labeled and added to THP‐1‐derived macrophages (THP‐1) in the presence or absence of 100 nM PROS1. Phagocytosis was quantified as the percentage of fluorescent THP‐1 containing fluorescently labeled Iono‐ or Control‐RBC. When Iono‐RBC were added to THP‐1 in the presence of PROS1, a 2‐fold increase in the proportion of fluorescent THP‐1 was observed (1.9 ± 0.3 + PROS1/‐PROS1 ratio, *n* = 5) compared to the condition in the absence of PROS1. In contrast, the percentage of fluorescent THP‐1 was similar in the presence and absence of PROS1 for control‐RBC (0.9 ± 0.2 + PROS1/‐PROS1 ratio, *n* = 5, Figure 1B). The percentage of fluorescent THP‐1 was very low (0.3% ± 0.3%) when cytochalasin D was added to inhibit phagocytosis (Figure 1C), while it was significantly increased from 19.5% ± 1.1% to 26.8% ± 1.6% (*p* < 0.05) and 31.9% ± 5.4% (*p* < 0.01) in the presence of 50 and 100 nM of PROS1 respectively (*n* = 3, Figure 1C). As we hypothesized that the prophagocytic effect of PROS1 would involve MerTK, we verified that a high proportion (86.7% ± 3.2%) of THP‐1 macrophages expressed MerTK (Figure 1D, *n* = 3). In the presence of UNC2025, a MerTK inhibitor, the percentage of fluorescent THP‐1 was reduced to 9.7% ± 1.7% (*p* < 0.01, *n* = 3) (Figure 1E). Hence, MerTK inhibition abolished the prophagocytic activity of PROS1. Here, non‐physiological Iono‐RBC were used for the proof of concept that PROS1 enhances the phagocytosis of PtdSer‐exposing RBC by THP‐1‐derived macrophages in a MerTK‐dependent manner. The effect of PROS1 on the phagocytosis of PtdSer‐exposing eryghosts was then assessed to evaluate the relevance of this novel PROS1 activity in the physiology of RBC clearance.

### 3.2 Protein S Enhances the Phagocytosis of Eryghosts Generated In Vitro in a MerTK‐Dependent Manner

**FIGURE 2:**
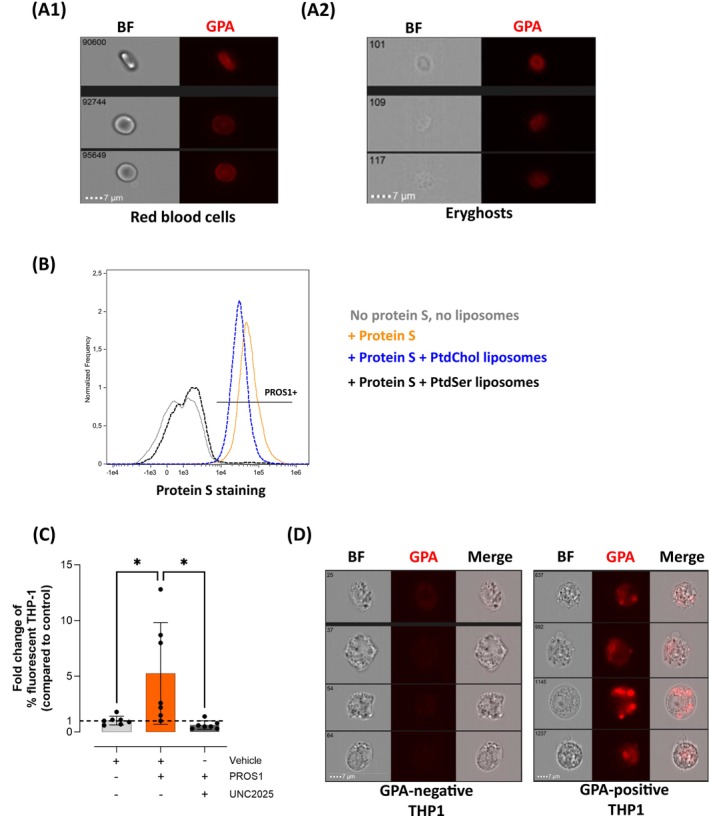
Protein S binds to phosphatidylserine on the surface of in vitro generated eryghosts and enhances their phagocytosis in a MerTK‐dependent manner. (A) Intact RBC (A1) and eryghosts produced in vitro (A2) visualized by imaging flow cytometry. Eryghosts are characterized by the combination of a ghost aspect in Brightfield (BF) and a positive anti‐glycophorin‐A (GPA) staining. (B) Binding of PROS1 to PtdSer the surface of eryghosts. Detection of PROS1 on the surface of in vitro‐generated eryghosts after being incubated with vehicle (light gray), PROS1 alone (orange), PROS1 pre‐incubated with 100 μM liposomes composed only of phosphatidycholine (PtdChol liposomes, blue) or 100 μM liposomes composed of 50% PtdChol and 50% PtdSer (PtdSer liposomes, black). A representative result from 3 independent experiments is shown. (C) In vitro phagocytosis of eryghosts in presence of vehicle (DMSO, light gray), PROS1 (100 nM), or PROS1 + UNC2025 (100 nM and 5 μM respectively, dark gray). The results are expressed as a ratio between the percentage of fluorescent THP‐1 macrophages in each condition described above and the percentage of fluorescent THP‐1 in the presence of culture medium alone. The black dotted line represents the absence of the prophagocytic effect (ratio = 1), *n* = 7, One‐way ANOVA with Tukey's multiple comparison tests were used, *p < 0.05. (D) Imaging flow cytometry images showing GPA‐negative (left panel) and GPA‐positive (right panel) THP‐1 macrophages after the phagocytosis assay. Multiple phagocytosed eryghosts are observed in GPA‐positive THP‐1 while none are detected in GPA‐negative THP‐1. [Color figure can be viewed at wileyonlinelibrary.com]

Hemolysis of normal RBC was triggered in vitro using a hypotonic lysis, and the cell suspension obtained was analyzed by imaging flow cytometry. A ghost aspect in brightfield associated with a positive anti‐GPA staining confirmed the transformation of normal RBC (Figure 2A1) into eryghosts (Figure 2A2). A high proportion of these eryghosts exposed PtdSer (53.8% ± 23.6%, *n* = 10). To confirm that PROS1 binds to PtdSer on the surface of eryghosts, liposomes were used as potential competitive inhibitors. PtdSer‐containing liposomes inhibited PROS1 binding on eryghosts, while control liposomes (100% phosphatidylcholine) did not affect this binding (Figure 2B). PROS1 significantly enhanced the phagocytosis of in vitro generated eryghosts by a 5.3 ± 4.6‐fold (*p* < 0.05 compared to vehicle, *n* = 7). Conversely, PROS1 did not increase the phagocytosis of eryghosts when the MerTK inhibitor UNC2025 was added (*p* = 0.95 compared to vehicle, *n* = 7, Figure 2C). Imaging flow cytometry confirmed that fluorescently labeled eryghosts were present in THP‐1 macrophages at the end of the in vitro phagocytosis assay (Figure 2D).

### 3.3 Eryghosts Circulate in the Peripheral Blood of Patients With SCD in Much Higher Proportions Than in Control Subjects

**FIGURE 3:**
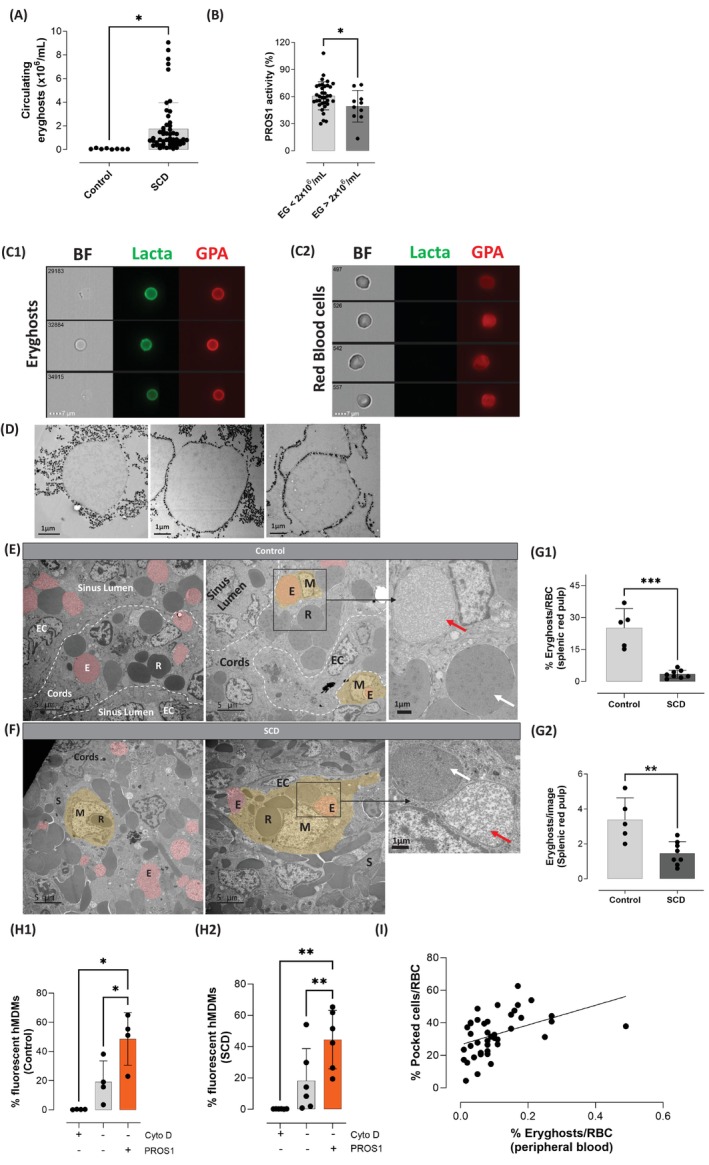
The higher proportion of circulating eryghosts in patients with sickle cell disease contrasts with the low proportion observed in the splenic red pulp and correlates with the intensity of hyposplenism. (A) Quantification of circulating eryghosts isolated from the platelet‐free plasma of healthy volunteers' (Control, *n* = 8) and patients with SCD (SCD, *n* = 53). Student *t*‐test is used as statistical test, *p < 0.05. (B) Activated protein C cofactor activity of PROS1 in patients with high (> 2 × 10^6^/mL, *n* = 10) and low circulating (< 2 × 10^6^/mL, *n* = 35) eryghost concentrations. Student *t*‐test is used as statistical test, *p < 0.05. (C1‐2) Circulating eryghosts (C1) and intact RBC (C2) from patients with SCD visualized by imaging flow cytometry after GPA and lactadherin staining (Lacta). Eryghosts are characterized by the combination of a ghost aspect in brightfield (BF) and a positive anti‐glycophorin‐A staining (GPA), and show a diffuse PtdSer exposition. (D) Representative TEM images of in vitro prepared eryghosts isolated using magnetic beads conjugated with Annexin V. Eryghosts appear as close‐to‐circular structures, of about 7 μm diameter, with internal granularity, surrounded by black particles (~50 nm diameter), which correspond to Annexin V‐conjugated magnetic beads. The eryghosts ultrastructure is similar in size and shape to that of the particles observed in the spleens sections presented in (E) and (F). (E, F) Representative TEM images showing spleen sections from (E) individuals without RBC disorder and (F) patients with SCD. Particles reminiscent of eryghosts are colored in red (a representative image was labeled with the letter **E**). Representative images of intact **R**BC with a homogeneous cytoplasm, or polymer‐containing RBC (**S** for **S**ickle), **E**ndothelial **C**ells, and **M**acrophages were labeled with the letters **R**, **S**, **EC** and **M**, respectively. The cytoplasm and nucleus of macrophages are colored in yellow. Phagocytosed eryghosts appear in orange. The delimitation between sinus lumens and cords by endothelial cell and basal fibers is represented by the white dotted line [26]. The area delineated by a black rectangle is magnified in the image on the right. The white and the red arrows indicate a RBC and an eryghost, respectively. (G1–G2) Quantification of eryghosts in the spleen of patients with SCD (*n* = 8) and controls (patients without RBC disorder who had undergone splenectomy, *n* = 5) with the following formula: %eryghosts = (number of eryghosts) × 100/[(number of eryghosts) + (number of intact RBC)] (G1) or as the number of eryghosts on each section image analyzed (G2). Student *t*‐test was used as statistical test, **p < 0.01, ***p < 0.001. (H1–H2) Phagocytosis of in vitro‐generated eryghosts by human monocyte‐derived macrophages (hMDMs) isolated from the whole blood of controls ( *n* = 4) (H1) or patients with SCD ( *n* = 6) (H2), in the presence of cytochalasin D (negative control, black), or culture medium (gray), or PROS1 (100 nM, orange). Paired one‐way ANOVA with Tukey's multiple comparison tests are used, * *p* < 0.05, ** *p* < 0.01. (I) Correlation between the percentage of eryghosts in the peripheral blood of patients with SCD (*n* = 44) and the splenic red blood cell filtration capacity defect assessed by the percentage of circulating pocked cells. A spearman test is used as a statistical test (*p* < 0.01, Spearman *r* = 0.44). [Color figure can be viewed at wileyonlinelibrary.com]

The concentration of circulating eryghosts was significantly increased (30‐fold) in patients with SCD (1.8 ± 2.2.10^6^/mL, *n* = 53) when compared to control subjects (0.06 ± 0.04.10^6^/mL, *n* = 8, *p* < 0.0001, Figure 3A). A marked heterogeneity was observed among patients with SCD. Patients with high eryghost concentrations (mean ± SD: 5.2 ± 2.2 × 10^6^/mL, *n* = 10) exhibited significantly lower PROS1 activity (49.3% ± 17.5%), a coagulation assay which reflects PROS1 levels, compared to those with a lower eryghost concentration (0.7 ± 0.4 × 10^6^/mL, *n* = 35; PROS1 activity: 60.8% ± 15.5%) as shown in Figure 3B. Moreover, the concentration of circulating eryghosts was positively correlated with hemolysis biomarkers such as the reticulocyte count (*p* < 0.05), lactate deshydrogenase (*p* < 0.01), and total bilirubin (p < 0.05), as shown in Figure S1. Circulating eryghosts were further characterized using imaging flow cytometry, showing a ghost aspect in brightfield, a positive GPA staining and a diffuse PtdSer‐exposure (Figure 3C1), as compared to intact RBC (Figure 3C2). Furthermore, when added exogenously, PROS1 bound circulating eryghosts (Figure S2).

### 3.4 Eryghosts Are Observed in the Splenic Red Pulp of Individuals Without RBC Disorders and Less Often in Patients With SCD

When observed by TEM, eryghosts produced in vitro after hypotonic lysis appear as cellular elements sharing size and morphology with RBC but mostly empty. The exposure of PtdSer at their surface was probed by the binding of Annexin V magnetic nanoparticles, as shown in Figure 3D. Morphologically similar cellular events reminiscent of eryghosts were frequently observed by TEM in spleen sections of individuals without RBC disorders (*n* = 5) who had undergone splenectomy, accounting for 25.1% ± 9.0% of all erythroid cells (Figures 3E and S3). These elements were observed in the red pulp, sometimes engulfed in macrophages together with intact RBC. Eryghosts were also observed in spleen sections of patients with SCD (*n* = 8) (Figures 3F and S3), although in a significantly smaller proportion compared to controls (3.4% ± 1.9% of all erythroid cells, Figure 3G1). This difference remained significant even after adjusting for erythrocyte congestion, with fewer eryghosts per surface unit in SCD patients compared to controls (3.4 ghosts/image in controls vs. 1.5 ghosts/image in SCD patients, *p* < 0.01, Figure 3G2).

### 3.5 Monocyte‐Derived Macrophages From SCD Patients Maintain PROS1‐Enhanced Phagocytosis of Eryghosts

An impaired phagocytic capability of the macrophages from patients with SCD could explain the higher proportion of eryghosts in circulation compared to controls. We performed a phagocytosis assay using human monocyte‐derived macrophages (hMDMs) isolated from patients with SCD (*n* = 6) and healthy controls (*n* = 4), in the presence or absence of PROS1. Following incubation with in vitro–generated eryghosts, baseline phagocytic activity was observed in both groups, with 18.2% ± 20.5% fluorescent hMDMs in healthy controls and 19.1% ± 14.4% in SCD patients (Figure 3H1,H2). Similarly to THP‐1‐derived macrophages, the percentage of fluorescent hMDMs was dramatically reduced upon treatment with cytochalasin D (Figure 3H). In contrast, the addition of PROS1 significantly enhanced phagocytic activity, increasing the proportion of fluorescent hMDMs to 48.6% ± 18.0% in healthy controls (*p* < 0.05, Figure 3H1) and 44.5% ± 18.7% in SCD patients (*p* < 0.01, Figure 3H2).

### 3.6 The Concentration of Circulating Eryghosts Correlates With the Severity of Hyposplenism in Patients With SCD

Another hypothesis is that eryghosts circulate more in patients with SCD due to defective RBC filtration in the spleen, reducing their retention in the splenic red pulp. This hypothesis is supported by a significant correlation between the concentration of circulating eryghosts and that of pocked cells, a specific biomarker of spleen filtration capacity (*r* = 0.44, *p* < 0.01, Figure 3I).

### 3.7 Circulating Eryghosts From Patients With SCD Are Procoagulant and Adhere to Endothelial Cells

**FIGURE 4:**
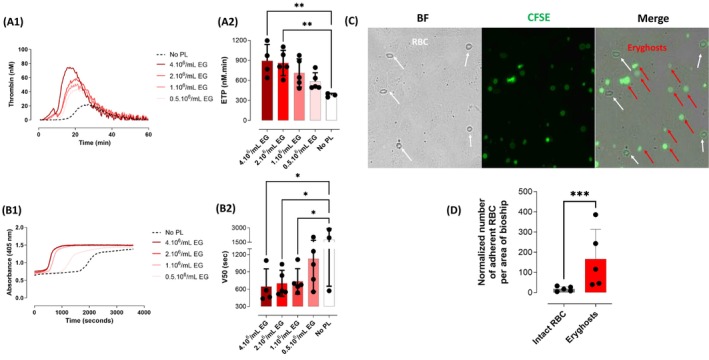
In patients with SCD, circulating eryghosts are procoagulant and adherent to endothelial cells. (A1) and (A2) Thrombin generation is measured in a normal plasma without phospholipids (No PL, *n* = 3), or increasing concentrations of circulating eryghosts isolated from patients with SCD (0.5–2.10^6^/mL, *n* = 5; and 4.10^6^/mL, *n* = 4). Coagulation is triggered by 1 pM tissue factor. (A1) Thrombin generation assay progress, data showed is a representative example. (A2) Endogenous thrombin potential (ETP) calculated in each condition: Without phospholipids (negative control condition, No PL, white bar), or with increasing concentrations of eryghosts from 0.5 to 4.10^6^/mL. **p < 0.01. (B1) and (B2) Fibrin formation is measured in a normal human plasma without phospholipids (No PL, *n* = 3), or increasing concentrations of circulating eryghosts (EG) isolated from patients with SCD (0.5–2.106/mL, *n* = 5; and 4.106/mL, *n* = 4). Coagulation is initiated by 1 pM tissue factor. (B1) Fibrin generation assay progress curves of one representative experiment (B2) V50 (time corresponding to half‐maximal absorbance at 450 nm) is calculated in each condition: Without phospholipids (negative control condition, No PL, white bar), or with increasing concentrations of eryghosts (EG) from 0.5 to 4.10^6^/mL. One‐way ANOVA with Dunnett's multiple comparison tests are used (control condition: No PL), *p < 0.05. (C) Adherent eryghosts and intact RBC on HMEC‐1. Intact RBC (white arrows) are detected in brightfield (BF) and CFSE+ images, while eryghosts (red arrows) are only detected in the CFSE+ images. (D) Normalized number of intact RBC (gray) and eryghosts (red) adherent to the activated endothelium, *n* = 5. Paired Student t‐test is used, ***p < 0.001. [Color figure can be viewed at wileyonlinelibrary.com]

The potential ability of eryghosts isolated from patients with SCD to support blood coagulation reactions was tested using a thrombin generation assay (TGA) and a fibrin formation assay. In normal plasma, isolated eryghosts promoted thrombin generation (Figure 4A1), increasing endogenous thrombin potential (ETP) values by 54.8%, 88.2%, 127.7%, and 136.4% when eryghosts were added at concentrations of 0.5, 1.0, 2.0, and 4.0.10^6^/mL, respectively (Figure 4A2). Likewise, isolated eryghosts accelerated fibrin formation in normal plasma by shortening the time corresponding to half‐maximal 450 nm absorbance (V50) (Figure 4B1). V50 values were decreased by 36.7%, 58.9%, 60.7%, and 63.9% after adding eryghosts concentrations of 0.5, 1.0, 2.0, and 4.0.10^6^/mL, respectively (Figure 4B2).

The dynamic adhesion of intact RBC or circulating eryghosts from five patients with SCD to activated microvascular endothelial cells was measured in a flow adhesion assay. Thus, mixtures containing approximately 50% of eryghosts and 50% of intact RBC were prepared and perfused on TNF‐α‐activated monolayers of HMEC‐1 cells. After sedimentation and washing steps, the normalized number of adherent eryghosts per area of biochip was increased by 8.3 ± 2.8‐fold when compared to intact RBC (166.1 ± 147.7 and 18.2 ± 11.9, respectively, *p* < 0.001, *n* = 5, Figure 4C,D). These results strongly suggest that eryghosts from patients with SCD are more adherent to microvascular endothelial cells than intact RBC.

### 3.8 In SCD, Protein S Stimulates MerTK‐Mediated Phagocytosis of Circulating Eryghosts

**FIGURE 5:**
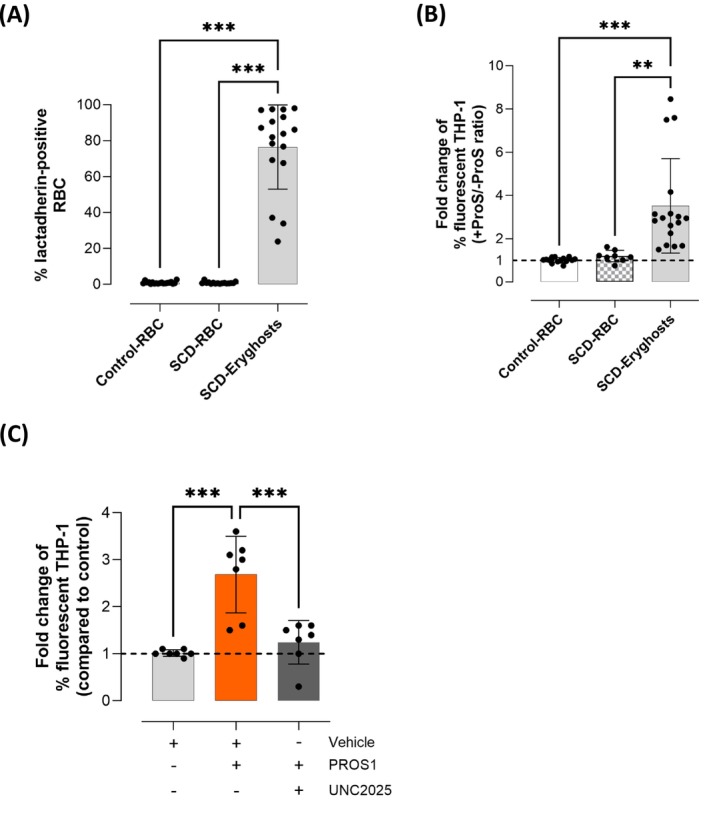
In patients with SCD, protein S enhances the phagocytosis of circulating eryghosts in a MerTK‐dependent manner, without affecting intact RBC. (A) Phosphatidylserine exposure on the surface of normal RBC (Control‐RBC, *n* = 19), intact RBC from patients with SCD (SCD‐RBC, *n* = 17), and circulating eryghosts from patients with SCD (Eryghosts, *n* = 17), quantified by flow cytometry after lactadherin‐FITC staining. Intact RBC are isolated from non‐transfused patients with SCD. One‐way ANOVA with Tukey's multiple comparison tests are used as statistical test***p < 0.001. (B) Phagocytosis of normal RBC (Control‐RBC, *n* = 14), intact patients with SCD' RBC (SCD‐RBC, *n* = 8), and circulating eryghosts from patients with SCD (Eryghosts, *n* = 17) in the presence or absence of PROS1. Results are expressed as the ratio between the percentage of fluorescent THP‐1 in the presence and in the absence of PROS1. The black dotted line represents the absence of the prophagocytic effect of PROS1 (ratio = 1). One‐way ANOVA with Tukey's multiple comparison tests are used as statistical test *p < 0.05, **p < 0.01. (C) Phagocytosis of circulating eryghosts in the presence of vehicle (DMSO, light gray) or PROS1 (100 nM, orange), or PROS1 (100 nM) and UNC2025 (5 μM, dark gray). The results are expressed as the ratio between the percentage of fluorescent THP‐1 macrophages in each condition described above and the percentage in the presence of culture medium alone. One‐way ANOVA with Tukey's multiple comparison tests were used, ***p < 0.001. [Color figure can be viewed at wileyonlinelibrary.com]

The impact of PROS1 on the phagocytosis of circulating eryghosts isolated from patients with SCD was characterized by flow cytometry. A high proportion of isolated eryghosts were PtdSer‐positive (76.4% ± 23.5%, *n* = 17), unlike Control‐RBC (0.9% ± 0.6%, *n* = 19) and patients with SCD's intact RBC (0.8% ± 0.6%, *n* = 16, Figure 5A). Phagocytosis of circulating eryghosts from patients with SCD was 3.5 ± 2.2‐fold higher in the presence of PROS1 (*n* = 17), whereas PROS1 did not increase the phagocytosis of intact RBC from patients with SCD (1.2 ± 0.3 + PROS1/‐PROS1 ratio, *n* = 8, Figure 5B) nor Control‐RBC (1.0 ± 0.1 + PROS1/‐PROS1 ratio, *n* = 14), consistent with the absence of PtdSer‐exposure. As for iono‐RBC and in vitro eryghosts, this effect was significantly inhibited when the MerTK inhibitor UNC2025 was added (Figure 5C).

## 4 Discussion

This study demonstrates that, as for nucleated cells, PROS1 enhances the phagocytosis of RBCs exposing high levels of PtdSer, creating a bridge between PtdSer and the MerTK receptor on macrophages. Notably, this mechanism contributes with high efficiency to the phagocytosis of PtdSer‐exposing eryghosts, a process recently shown to be involved in the physiological clearance of RBC [10]. PROS1‐dependent phagocytosis of eryghosts is likely protective in SCD, by enhancing the clearance of a pathogenic cell subpopulation prone to adhere to endothelial cells and locally trigger the coagulation cascade. Uncovering this new role for PROS1 provides a deeper understanding of SCD pathophysiology and opens the way to the identification of new therapeutic targets.

To our knowledge, this study is the first to investigate the impact of PROS1 on erythrophagocytosis. Previous research has explored the role of Gas6, a protein sharing high homology with PROS1 that also bridges PtdSer to TAM receptors (in particular Axl), in relation to PtdSer‐exposing RBC clearance in mice [9]. Mice deficient in Gas6 (*Gas6−/−*) exhibited impaired clearance of PtdSer‐exposing RBC. This observation is consistent with our results, suggesting that PROS1 and Gas6 may have a redundant role in this cellular effect, as described for other TAM‐mediated processes including apoptotic cells clearance [30]. A similar study using *PROS1−/−* mice could not be conducted, since complete PROS1 deficiency is incompatible with life in mice, as opposed to Gas6 deficiency. Future research could evaluate erythrophagocytosis in *MerTK−/−* mice. Given the functional redundancy between PROS1 and Gas6, studying *MerTK−/− Axl−/−* double knockout mice along with single mutants could provide a broader understanding. Notably, *MerTK−/−* or *Axl−/−* single mutants failed to reveal the accumulation of non‐engulfed apoptotic cells as opposed to *MerTK−/− Axl−/−* double knockout mice [31].

As previous reports showed that eryghosts are prone to recognition by splenic macrophages [10] and exposed PtdSer [12, 13], we focused on this RBC subpopulation to evaluate the relevance of PROS1 in erythrophagocytosis. Adding to this previous work [10], we found by TEM apparently empty elements, similar to RBC in size and shape, in sections of the splenic red pulp of individuals without RBC disorder and of patients with SCD. These elements were morphologically similar to eryghosts produced in vitro and observed by TEM in this study, and to eryghosts visualized by TEM [32] and cryo‐EM [12] in previous studies, strongly suggesting that these former particles are eryghosts. It remains uncertain whether eryghosts form in situ from retained RBC that burst in the red pulp, as proposed by Klei et al. [10], or result from intravascular hemolysis and are subsequently trapped in the spleen, as suggested by the presence of circulating eryghosts in the context of SCD. We showed that PROS1 significantly enhanced the phagocytosis of eryghosts in a MerTK‐dependent manner. As MerTK is highly expressed in RPM [33], the PROS1/MerTK axis likely participates in RBC turnover, both in physiology and in hemolytic anemia.

In contrast with a previous work [10], we observed intact RBC in splenic red pulp macrophages, along with eryghosts, both in normal and in SCD spleens. This indicates that engulfment of eryghosts is not the only mechanism leading to erythrophagocytosis in the spleen, consistent with other mechanisms previously described. In senescent RBCs, the protein band 3 undergoes oxidative modifications leading to complement‐mediated clearance [34, 35, 36]. CD47, a “don't eat me” signal, can undergo a conformational change under oxidative stress, promoting a pro‐phagocytic signal [37, 38]. Desialylation of membrane proteins may also contribute to erythrophagocytosis [39]. More recently, Ningtyas et al. suggested that platelets may participate in erythrophagocytosis through the formation of RBC–platelet aggregates [40]. Of note, PROS1 could also be involved in the latter mechanism, as platelets involved in these aggregates expose PtdSer.

We did not find any increased PtdSer exposure in intact RBC from patients with SCD by flow cytometry. This result contrasts with previous observations [41, 42, 43, 44, 45] but is in keeping with a recent phenotypic analysis of RBC in SCD [13]. In the same study, two PtdSer‐exposing RBC subpopulations were observed by imaging flow cytometry: eryghosts with a diffuse exposure and intact RBC with intense punctate dots. In our study, PROS1 had no effect on the phagocytosis of intact RBC from patients with SCD but showed a prophagocytic effect on eryghosts. Surprisingly, PROS1 could not be detected on the surface of circulating eryghosts under the tested conditions (data not shown). However, the binding of exogenous purified plasma‐derived PROS1 added to eryghosts was visualized. PROS1 might have detached from eryghosts during sampling or the monoclonal antibody may not recognize modified PROS1 because of oligomerization or oxidation due to its binding to PtdSer [46]. More likely, PROS1‐opsonized eryghosts were very efficiently trapped in the spleen, leaving the remaining circulating eryghosts unopsonized. Similarly, the removal of apoptotic cells is very efficient [47].

We found a significantly higher proportion of circulating eryghosts in the peripheral blood of patients with SCD compared to healthy individuals, although with marked inter‐patient heterogeneity. Lower PROS1 levels were observed in the patients with SCD exhibiting the highest eryghost concentrations. These findings suggest that PROS1 deficiency may contribute to impaired clearance of eryghosts in SCD. As PROS1 levels are frequently reduced in patients with SCD, our results also raise the possibility that this deficiency may result, at least in part, from its consumption during PROS1‐dependent erythrophagocytosis. However, additional mechanisms are likely to underlie both observations. On the one hand, the increased proportion of circulating eryghosts in patients with SCD could result from their increased generation by sustained intravascular hemolysis, as suggested by the correlation between this proportion and biomarkers of hemolysis (reticulocyte count, LDH, and total bilirubin). Moreover, functional hyposplenism may also contribute to the insufficient elimination of circulating eryghosts by the spleen, as suggested by the correlation between circulating eryghosts and pocked cells. On the other hand, additional mechanisms were proposed to underlie PROS1 deficiency in SCD, such as consumption due to chronic coagulation activation [16], impaired hepatic synthesis related to liver dysfunction or vitamin K deficiency [48], and hypoxia [49].

**FIGURE 6:**
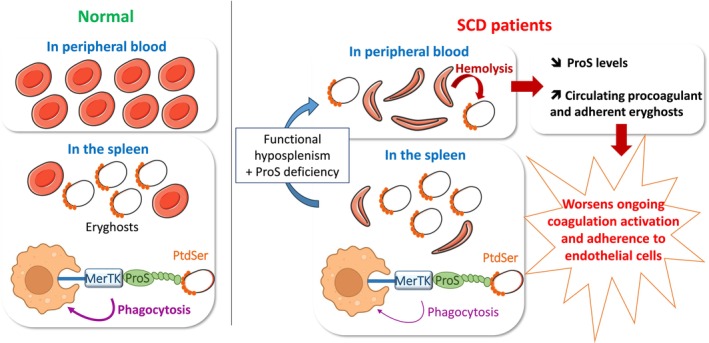
Proposed model for the implication of PROS1 in erythrocyte turnover and in the pathophysiology of sickle cell disease. In healthy individuals (normal), eryghosts are almost absent from the peripheral blood but present in the spleen, where these particles are removed by a PROS1‐promoted phagocytosis process. In patients with SCD, intravascular hemolysis promotes eryghosts formation in peripheral blood, and functional hyposplenism and PROS1 deficiency decrease their splenic clearance. As a consequence, eryghosts accumulate in circulation and may participate in the SCD pathophysiology by promoting coagulation activation and adherence to endothelial cells. Abbreviations: SCD sickle cell disease, PtdSer Phosphatidylserine, PROS1 Protein S. [Color figure can be viewed at wileyonlinelibrary.com]

Removing circulating PtdSer‐exposing macrovesicules was shown before to be associated with lengthened clotting times in individuals with SCD [14]. In this study, we investigated more precisely the impact of increasing concentration of eryghosts on coagulation in SCD and showed increased thrombin generation and fibrin formation. More importantly, we reported for the first time that eryghosts are prone to adhere to the vascular endothelium, a crucial feature in the pathophysiology of SCD. As such, eryghosts could contribute to the generation of thrombin at the endothelial surface thereby triggering or exacerbating vaso‐occlusive events [50, 51, 52]. Acquired PROS1 deficiency may therefore create a pathogenic loop in SCD by worsening coagulation activation and decreasing the clearance of these pathogenic cell remnants. This mechanistic hypothesis is illustrated in Figure 6.

The pathogenic role of eryghosts in SCD might be extended to other hemolytic anemias and to splenectomized patients. Previous studies have indicated that splenectomized patients face a higher risk of thromboembolic events [53], which is further elevated if splenectomy is associated with ongoing hemolysis [54]. Impaired clearance of eryghosts in splenectomized patients, coupled with an increased production of these cell remnants in patients with ongoing hemolysis, may explain these observations. Further studies evaluating circulating and intrasplenic eryghosts together with PROS1 levels in these distinct patient populations are warranted.

Finally, these results could pave the way for new treatment strategies in SCD. We showed that macrophages from patients with SCD retain a phagocytic activity that is boosted by PROS1. Therefore, the PROS1‐dependent erythrophagocytosis could be an interesting new target in this disease. We recently identified a llama‐derived anti‐PROS1 single‐domain antibody, named PS003biv, which enhances the APC‐cofactor activity of PROS1 and has antithrombotic effects while sparing physiological hemostasis in mice [55]. PS003biv targets the SHBG‐like domain of PROS1, which interacts with MerTK. Hence, PS003biv could be a promising new treatment option by promoting not only the anticoagulant activity but also the prophagocytic effect of PROS1 on circulating eryghosts.

## Author Contributions

C.A. and A.F. performed research, analyzed and interpreted data, and wrote the manuscript. R.G., E.B., C.G., T.T.‐M., S.M., and S.A. performed research, analyzed and interpreted data. Z.C., M.K., S.M., and A.L. collected data. M.D., M.M., A.S., S.E., and P.R. contributed vital analytical tools. L.C., S.L., V.B., and E.B. contributed vital reagents. P.B., A.B., L.J., and P.A. designed research; D.B., F.S., and C.R. initiated, designed and supervised research, analyzed and interpreted data, and wrote the manuscript. All authors revised the manuscript.

## Ethics Statement

Human subjects were included at two hospitals of the Assistance Publique‐Hôpitaux de Paris (AP‐HP) network in a study entitled “Pathophysiological Explorations of Red Blood Cells” (ClinicalTrials.gov reference NCT03541525). Human spleens were retrieved in the context of the Spleenvivo project approved by the “Ile‐de‐France II” Institutional Review Board on September 4, 2017 (#CPP 2015‐02‐05 SM2 DC).

## Conflicts of Interest

L.J. received consulting fees from Vertex; D.B. and F.S. received a research grant from CSL Behring. All the other co‐authors have no conflicts of interest to declare.

## Supporting information


**Figure S1:** Correlation between hemolysis biomarkers and the concentration of circulating eryghosts in patients with SCD. Correlation of circulating eryghosts with (A) the reticulocyte count (*n* = 44n *p* < 0.01, Spearman *r* = 0.47), (B) lactate deshydrogenase (*n* = 40, *p* < 0.01, Spearman *r* = 0.42), (C) total bilirubin (*n* = 44, *p* < 0.05, Spearman *r* = 0.33). A spearman test is used as a statistical test.

**Figure S2:** Imaging flow cytometry of eryghosts from the peripheral blood of patients with SCD after incubating with human plasma‐derived protein S. Eryghosts from the peripheral blood of patients with SCD (*n* = 3) are incubated with a physiological concentration of PROS1 (100 nM). Then, PROS1 (yellow) is probed on the surface of eryghosts along with GPA (red) and PtdSer (lactadherin staining, green).

**Figure S3:** Raw and colorized TEM images of splenic red pulp sections. (A) raw and colorized TEM images of sections from the spleen of individuals without RBC disorder; (B) raw and colorized TEM images of sections from the spleen of a patient with SCD.

**Data S1:** ajh70117‐sup‐0004‐supinfo.docx.

## Data Availability

Data are available upon reasonable request to the corresponding author.
