# Supplementary figures and images for: Protein S Enhances the Phagocytosis of Phosphatidylserine‐Exposing Erythrocytes: Implications in Sickle Cell Disease
Source: Am J Hematol. 2025 Oct 25;101(1):26–40. doi: 10.1002/ajh.70117 (PMC12669952; doi:10.1002/ajh.70117)

**(A)**

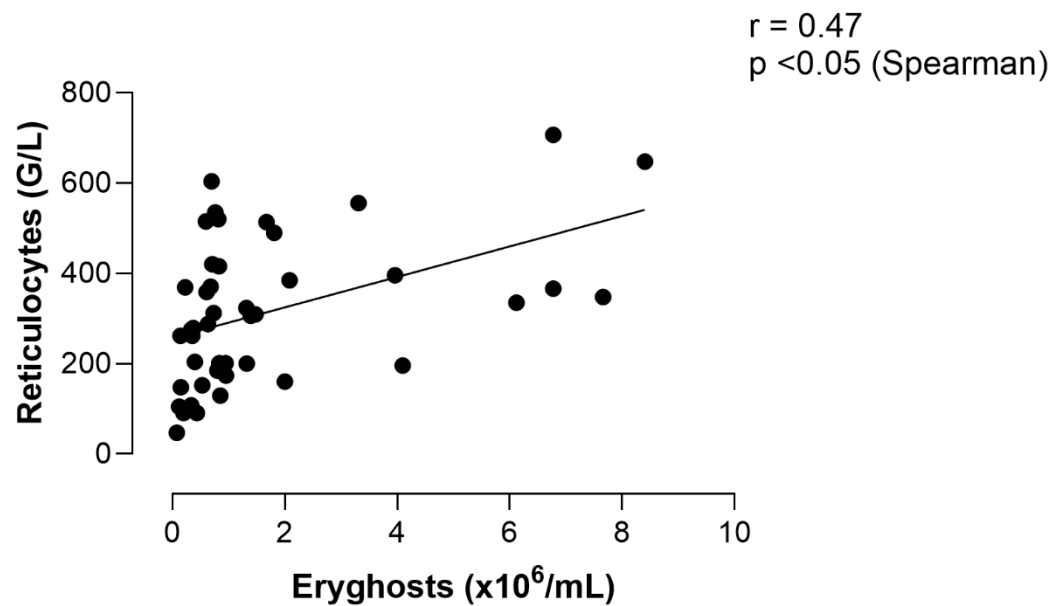

**(B)**

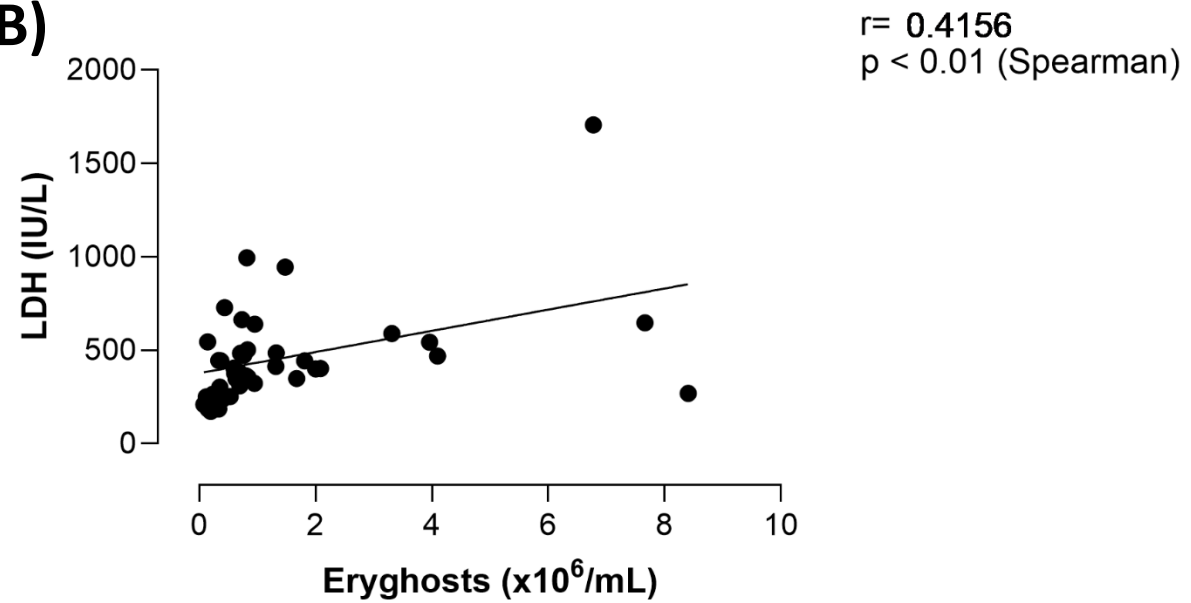

**(C)**

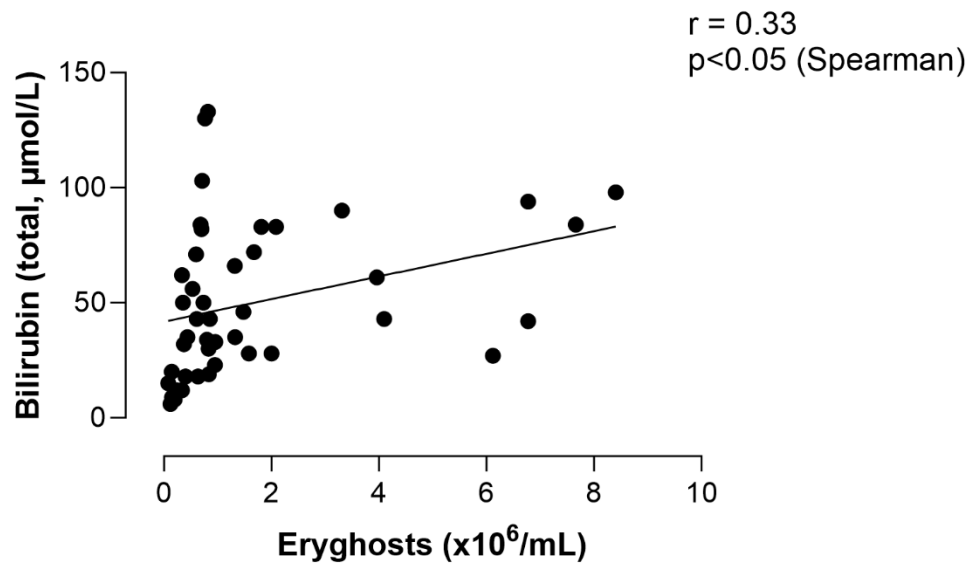

Supplement: Supplementary file 1 — Figure S1: Correlation between hemolysis biomarkers and the concentration of circulating eryghosts in patients with SCD. Correlation of circulating eryghosts with (A) the reticulocyte count (n = 44n p < 0.01, Spearman r = 0.47), (B) lactate deshydrogenase (n = 40, p < 0.01, Spearman r = 0.42), (C) total bilirubin (n = 44, p < 0.05, Spearman r = 0.33). A spearman test is used as a statistical test. [file AJH-101-26-s002.pdf]

**BF**

**Lacta**

**ProS**

**GPA**

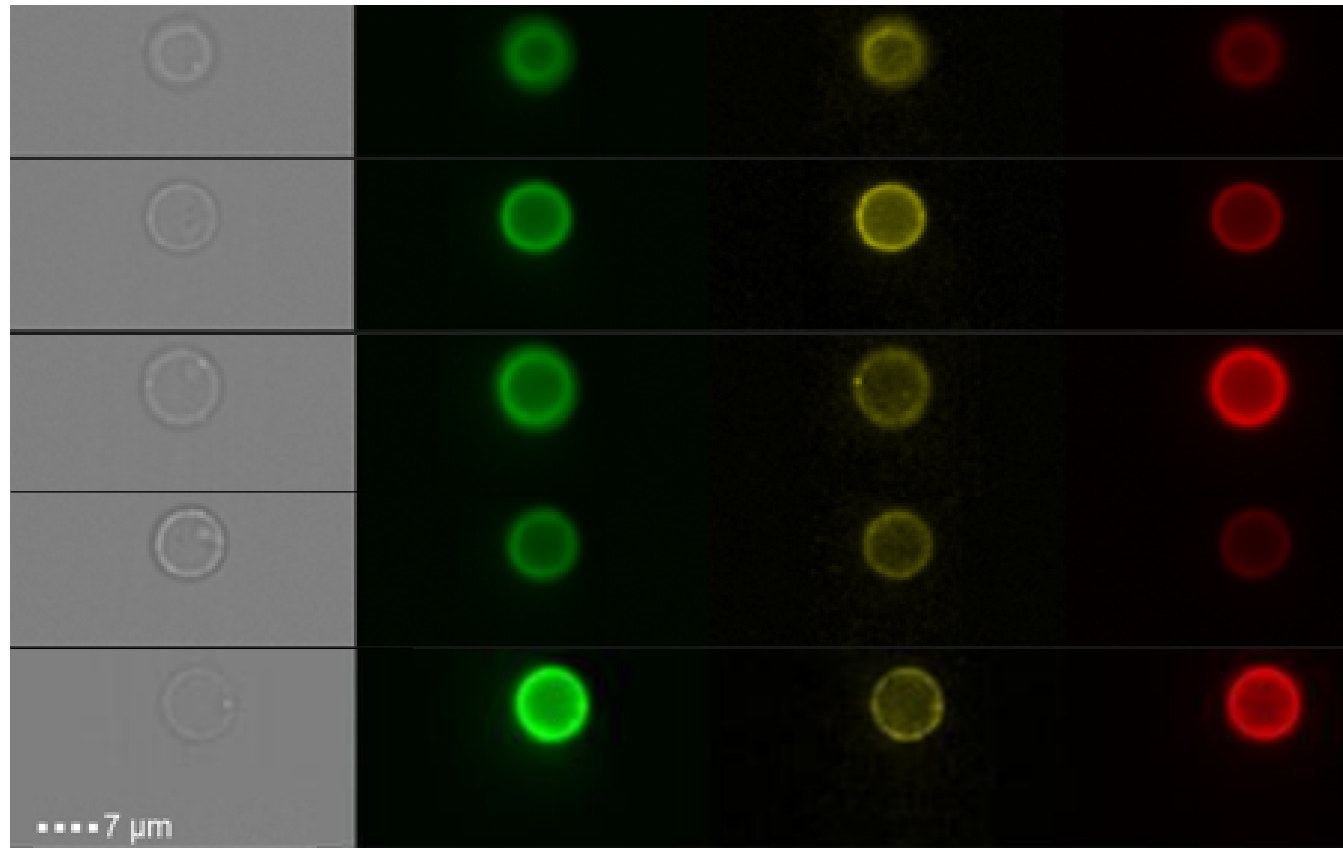

Supplement: Supplementary file 2 — Figure S2: Imaging flow cytometry of eryghosts from the peripheral blood of patients with SCD after incubating with human plasma‐derived protein S. Eryghosts from the peripheral blood of patients with SCD (n = 3) are incubated with a physiological concentration of PROS1 (100 nM). Then, PROS1 (yellow) is probed on the surface of eryghosts along with GPA (red) and PtdSer (lactadherin staining, green). [file AJH-101-26-s001.pdf]

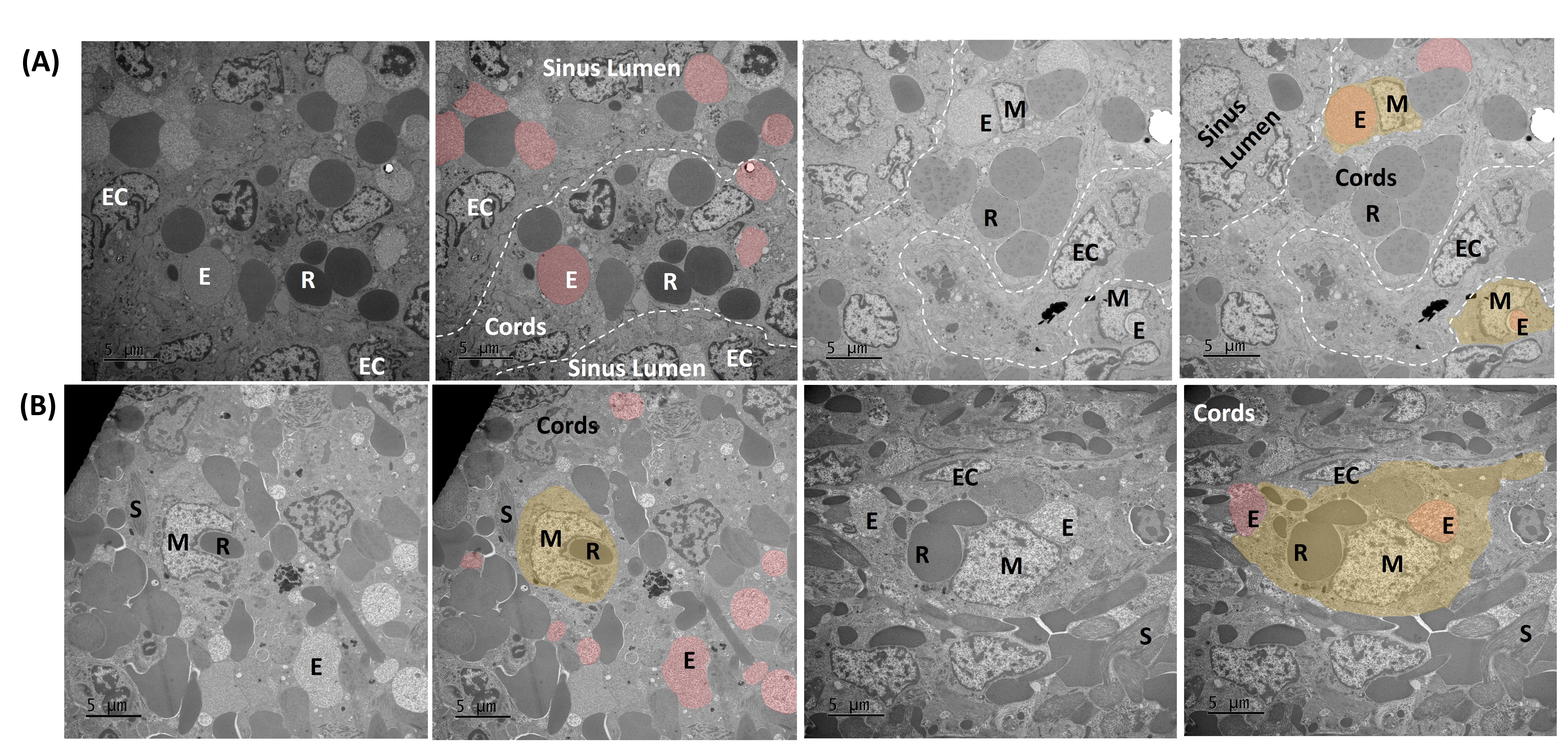

Supplement: Supplementary file 3 — Figure S3: Raw and colorized TEM images of splenic red pulp sections. (A) raw and colorized TEM images of sections from the spleen of individuals without RBC disorder; (B) raw and colorized TEM images of sections from the spleen of a patient with SCD. [file AJH-101-26-s004.jpg]
