# Supplementary material for: Protein S Enhances the Phagocytosis of Phosphatidylserine‐Exposing Erythrocytes: Implications in Sickle Cell Disease
Source: Am J Hematol. 2025 Oct 25;101(1):26–40. doi: 10.1002/ajh.70117 (PMC12669952; doi:10.1002/ajh.70117)
Supplement: Supplementary file 4 — Data S1: ajh70117‐sup‐0004‐supinfo.docx. [file AJH-101-26-s003.docx]

**SUPPLEMENTAL METHODS**

| Experiment | Reagent | Reference | Source | Final concentration or dilution |
| --- | --- | --- | --- | --- |
| Phosphatidylserine staining | FITC-labeled bovine lactadherin | 9-BLAC-FITC | Cryopep | 1:25 |
| Ionomycin treatment | Ionomycin | I3909 | Sigma Aldrich | 1µM |
| Anti-GPA staining | Alexa Fluor ^®^ 647-labeled anti-GPA antibody | FAB1228R | Bio-techne | 1:200 |
| Protein S staining | Anti-protein S antibody | MA1-40205 | Invitrogen | 1:50 |
|  | Alexa Fluor® 568-conjugated goat anti-Rat IgG | A11077 | Invitrogen | 1:500 |
|  | Human protein S purified from plasma | 90760 | Enzyme Research Laboratories | 100 nM |
|  | HBSS | 14025-092 | Invitrogen | N.A. |
| Quantification of eryghosts in the platelet-poor plasma | Fluorescent microbeads | Ultra Rainbow Fluorescent Particle 10.2 µm | CliniSciences | N.A. |
|  | Alexa Fluor ^®^ 647-labeled anti-GPA antibody | FAB1228R | Bio-techne | 1:200 |
| Phagocytosis assay | PMA | P1585 | Sigma Aldrich | 20 ng/mL |
|  | CFSE | 21888 | Sigma Aldrich | 20 µM |
|  | Alexa Fluor ^®^ 647-labeled anti-GPA antibody | FAB1228R | Bio-techne | 1:200 |
|  | Culture medium  RPMI 1640 + GlutaMAX™+ 25 mM Hepes | 72400-021 | Gibco | N.A. |
|  | Fetal bovine serum | 500105A1A | Dominique Dutscher | 10% |
|  | Penicillin-streptomycin | 15140122 | Gibco | 1% |
|  | Trypsin-EDTA | 25300054 | Gibco | 0.05% |
|  | Human protein S purified from plasma | 90760 | Enzyme Research Laboratories | 50-100 nM |
|  | UNC2025 | 16613 | Cayman Chemical | 5µM |
|  | Cytochalasin D | C2618 | Sigma Aldrich | 50 µg/mL |
|  | Ficoll Paque | 17-5442-03 | GE Healthcare | N.A. |
|  | CD14 microbeads | 130-050-201 | Miltenyi Biotec | 10µL for 10.10^6^ cells |
|  | M-CSF | 130-096-491 | Miltenyi Biotec | 50 ng/mL |
|  | Accutase | A6964 | Sigma-aldrich | Undiluted |
| MerTK expression | Anti-MerTK antibody | FAB8912A | Bio-techne | 1:10 |
|  | Control isotype Mouse IgG2b APC | IC0041A | Bio-techne | 1:10 |
| Thrombin generation assay | Normal human plasma | Cryocheck  CCN-10 | Cryopep | N.A. |
|  | PRP-reagent® | 86196 | Stago | 1:5 |
|  | Thrombin calibrator | 86192 | Stago | 1:5 |
|  | FluCa®Kit | 86197 | Stago | 1:6 |
| Fibrin formation assay | Normal human plasma | Cryocheck  CCN-10 | Cryopep | N.A. |
|  | Tissue factor | Dade Innovin^®^ B4212-41 | Siemens | 1/5000 |
| Multiple experiments | Bovine serum albumin | AlbuMAX  11021-037, | Gibco | 1% |
|  | Dulbecco’s Phosphate buffer saline  DPBS | 11875093 | Gibco | N.A. |

N.A. not applicable

**Preparation of ionomycin-treated RBC**

RBC at 0.4% hematocrit in Ringer solution containing 125 mM NaCl, 5 mM KCl, 1 mM MgSO_4_, 32 mM HEPES, and 1 mM CaCl_2_ were incubated with 1 µM ionomycin (I3909, Sigma Aldrich) for 1 hour (h) at 37°C, washed three times and resuspended in Ringer solution.

**Quantification of circulating eryghosts**

For the quantification of eryghosts in platelet-free plasma (PFP), Alexa Fluor® 647-conjugated anti-GPA antibody was added to the thawed, non-centrifuged PFP of patients with SCD or healthy volunteers. The quantification was performed by flow cytometry using fluorescent microbeads.

Eryghosts were quantified in the whole blood of patients with SCD using imaging flow cytometry as a percentage of total RBC after an anti-GPA satining with Alexa Fluor® 647-conjugated anti-GPA antibody.

**Binding of protein S to phosphatidylserine on the surface of *in vitro*-generated eryghosts**

To demonstrate the binding of PROS1 to PtdSer on the surface of eryghosts, a competition assay using liposomes was performed as previously described^1^. In brief, liposomes (100 µM) consisting of either 100% phosphatidylcholine (PtdChol) or a 50:50 mixture of PtdChol and PtdSer (prepared by membrane extrusion^2^) were incubated with PROS1 (100 nM) at 37°C for 15 minutes. These mixtures were then added to eryghosts for 15 min at 37°C. As a positive control, PROS1 (100 nM) was added to eryghosts without liposomes. For the negative control, neither PROS1 nor liposomes were added. PROS1 binding was assessed in all the conditions by imaging flow cytometry (reagents used for PROS1 staining are listed in the table above).

**Automated assays**

*Activated protein C cofactor activity of PROS1*

The APC-cofactor activity of PROS1 was measured using a clotting based assay on ACL-TOP intrument (STACLOT® PS, Stago, Asnières-sur-Seine, France) on ACL-TOP instruments (Werfen, Bedford, MA, USA).

*Reticulocyte count*

The reticulocyte count was performed on a Sysmex XN (Kobe, Japan) instrument.

*Lactate deshydrogenase*

Lactate dehydrogenase (LDH) levels were determined using an enzymatic method on an Abbott Alinity platform.

*Total bilirubin*

Total bilirubin was measured using a colorimetric method based on diazonium ion reaction on an Abbott Alinity analyzer.

**Transmission electron microscopy**

*Images of spleen sections*

Ultra-thin sections (90 nm) of these blocks were obtained with a Leica EM UC7 ultramicrotome (Wetzlar, Germany). Sections were stained with 2% uranyl acetate (Agar Scientific), 5% lead citrate (Sigma), and observations were made with a transmission electron microscope (JEOL 1011, Tokyo, Japan).

*Identification of macrophages in spleen sections*

Macrophages were characterized by light to slightly dense cytoplasm and small nucleocytoplasmic ratio, a nucleus with dense and sparse chromatin at the periphery (interspersed with light area), the presence of hypo- and hyper-dense vesicles in a sparkling and heterogenous cytoplasm, presence of pseudopods, pinocytosis vacuoles, and absence of fibers in the cytoplasm.

*Images of eryghosts prepared in vitro*

Eryghosts aggregates were successively fixed, dehydrated and embedded in Epon-Araldite. Thin sections were stained successively with 5% uranyl acetate and 1% lead citrate. TEM observations were performed with a FEI CM120 electron microscope (FEI, USA) operated at 120 kV. Images were recorded with a USC1000 slow scan CCD camera (Gatan, USA).

**Procoagulant effects of circulating eryghosts from patients with SCD**

Frozen PFP from patients with SCD were thawed for 5 min at 37°C, gently mixed, and further centrifuged (at 3000 g for 15 min). The pellets containing circulating eryghosts were suspended in 50 µL of a Hepes/BSA buffer (20 mM Hepes, 6% BSA, pH 7,4).

*Thrombin generation assay*

Calibrated automated thrombography-based thrombin generation assays (TGA, Stago) were run in 96-well plates. Before analysis, 80 µL of normal human plasma was spiked with either 10 µL of Hepes/BSA buffer or increasing concentrations of circulating eryghosts diluted in Hepes/BSA buffer (0.5-4.10^6^/mL). Then, the spiked plasma was mixed with 20 µL of PRP-reagent® (containing tissue factor and minimal amounts of phospholipids) except for calibration wells, for which plasma was mixed with 20 µL of thrombin calibrator. The plate was further incubated for 10 min at 37°C before triggering thrombin generation with 20 µL of fluorescent substrate diluted in calcium-containing buffer (FluCa®Kit). Thrombin generation curves were analyzed, and TGA variables (i.e., the lag time, endogenous thrombin potential (ETP), thrombin peak, and time-to-peak were calculated using Thrombinoscope software.

*Fibrin formation assay*

The fibrin formation assay was performed with normal human plasma in 96-well half-area microplates. Each well was prefilled with 60 μL of plasma. Different trigger mixes were prepared: (i) tissue factor + 25 mM CaCl_2_ + Hepes/BSA and (ii) tissue factor + 25 mM CaCl_2_ + increasing concentrations of circulating eryghosts diluted in Hepes/BSA buffer (0.5-4.10^6^/mL). Plasma and trigger mixes were prewarmed at 37°C for 5 min. Fibrin formation was initiated by adding 30 μL of trigger mix to the plasma. The samples were shaken for 10 seconds, and the absorbance at 405 nm was read every 8 seconds for 30 min at 37 °C using a microplate reader (MP96, Safas). The clotting time was defined as the time needed to reach the midpoint between the minimum and maximum absorbance at 405 nm (V50).

***In-vitro* dynamic adhesion assay of RBC and eryghosts to endothelial monolayers stimulated with TNF-α**

Mixtures containing RBC and eryghosts were stained with CFSE (20 µM) and diluted in HBSS-5 mM CaCl_2_-1% albumin. Then, these mixtures were perfused for 10 min at a shear stress of 0.2 dyn/cm^2^ using the ExiGo™ pump (Cellix Ltd., Dublin, Republic of Ireland) in the Vena8 Endothelial+ biochip (Cellix Ltd.). After a 15-min sedimentation step, washes were performed for 10 min at 0.2 dyn and 5 min at 1 dyn with HBSS-5 mM CaCl_2_-1% albumin to remove non-adherent RBC and eryghosts. Finally, 17 representative areas were captured along the centre line of the channels using the AxioObserver Z1 microscope (20X objective) and Zen blue 3.1 software (Carl Zeiss). Images were analyzed using ImageJ software (Rasband, WS, ImageJ, US National Institutes of Health, Bethesda, Maryland, USA) to count adherent RBC and eryghosts.

1. Anderson HA, Maylock CA, Williams JA, Paweletz CP, Shu H, Shacter E. Serum-derived protein S binds to phosphatidylserine and stimulates the phagocytosis of apoptotic cells. *Nat Immunol*. 2003;4(1):87-91. doi:10.1038/ni871

2. MacDonald RC, MacDonald RI, Menco BPhM, Takeshita K, Subbarao NK, Hu L rong. Small-volume extrusion apparatus for preparation of large, unilamellar vesicles. *Biochimica et Biophysica Acta (BBA) - Biomembranes*. 1991;1061(2):297-303. doi:10.1016/0005-2736(91)90295-J
